# The *Escherichia coli* uropathogenic-specific-protein-associated immunity protein 3 (Imu3) has nucleic acid -binding activity

**DOI:** 10.1186/1471-2180-14-16

**Published:** 2014-01-28

**Authors:** Miha Črnigoj, Zdravko Podlesek, Maruška Budič, Darja Žgur-Bertok

**Affiliations:** 1 Department of Biology, Biotechnical Faculty, University of Ljubljana, Ljubljana, Slovenia

**Keywords:** *Escherichia coli*, Imu3, Immunity protein, Uropathogenic-specific protein DNA/RNA binding

## Abstract

**Background:**

The *Escherichia coli* uropathogenic-specific protein (Usp) is a bacteriocin-like genotoxin, active against mammalian cells and associated with *E. coli* strains that provoke pyelonephritis, prostatitis and bacteraemia. Usp is encoded by a small pathogenicity island with three downstream small open reading frames (Imu1-3) that are believed to provide immunity to the producer. To prevent host suicide, colicins, bacteriocins of *E. coli*, form tight complexes with their cognate immunity proteins. Colicin – immunity protein complexes are among the strongest protein complexes known. Here, the Usp associated immunity protein 3 (Imu3) was partially characterized to gain insight into its role and mechanism of activity.

**Results:**

Isolation and partial characterisation of the Usp-associated immunity protein-3 (Imu3) revealed that, while Usp and Imu3 do not form a high affinity complex, Imu3 exhibits DNA and RNA binding activity. Imu3 was also shown to protect DNA against degradation by colicin E7.

**Conclusions:**

Our data infer that nonspecific DNA binding of the Imu3 immunity protein, prevents suicide of *E. coli* producing the genotoxin Usp.

## Background

The *Escherichia coli* uropathogenic-specific protein (Usp) has been shown to be associated with *E. coli* strains that provoke pyelonephritis, prostatitis and bacteraemia, and with increased virulence and fitness of pathogenic strains of *E. coli*[1-4]. Nucleotide sequence analysis has shown approximately 45% sequence identity of the Usp C-terminal region with that of the *E. coli* bacteriocin colicin E7, which has nuclease activity, while the Usp N-terminal region is similar to the Type VI protein secretion system component (Hcp like) [5-7]. It has been proposed that Usp acts as a bacteriocin against competing *E. coli* strains and that it also enhances infectivity in the urinary tract. Recently, we demonstrated the genotoxic activity of Usp against mammalian cells [5,8]. To protect the colicin-producing cell from its own toxin, colicin-encoding operons generally harbour one cognate immunity gene [9]. Colicins and their immunity proteins have some of the strongest protein-protein affinities, which result in the formation of stable colicin–immunity protein complexes [10,11]. In contrast, downstream of the *usp* gene, there are three short open reading frames designated *orfU1-3* (288, 294, and 291 bp long, respectively), that are believed to be involved in the protection of the Usp-producing cell from its own nuclease activity [5]. The immunity proteins coded by the *usp* gene operon have a characteristic two-histidine region which appears to enable the inactivation of the Usp DNase activity [10]. However, Usp-encoding strains that do not have all three *orfU* immunity protein genes have been described. All three immunity proteins are thus not essential for the protection of the Usp producers, although Usp is lethal when it is expressed alone in *E. coli.* It has been postulated that none of the three proteins is exclusively required for Usp protein synthesis [6]. As protection of the Usp-producing bacterial cell might be provided by a mechanism that is different from that of the colicins, we have investigated the *E. coli* Usp-associated immunity protein Imu3, previously designated OrfU3. Our study indicates that Imu3 has protective non specific DNA-binding abilities that could have possible biotechnological potential.

## Results and discussion

**Figure 1:**
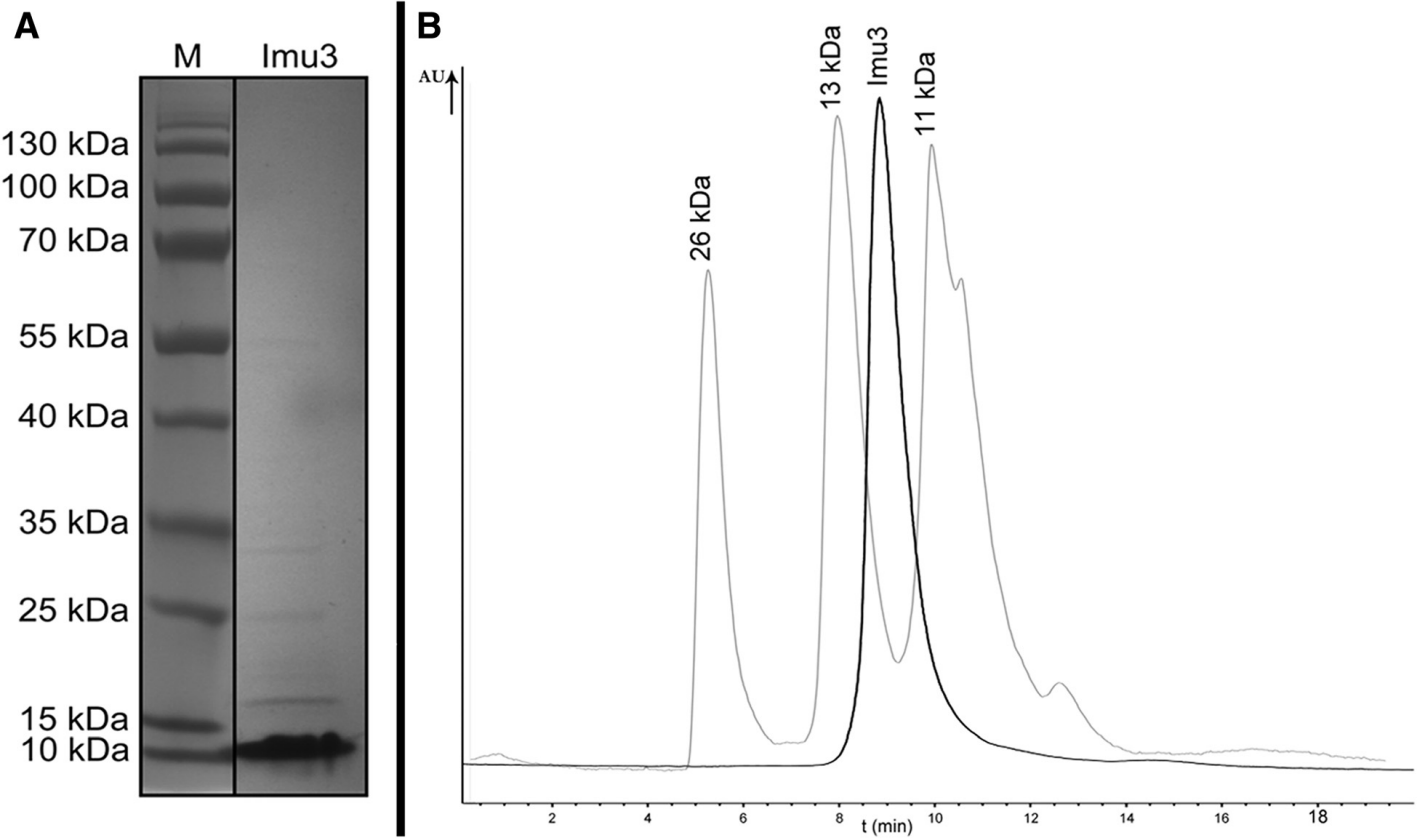
**Purified Imu3 protein. (A)** SDS PAGE gel of Imu3 isolated using Ni-NTA agarose affinity chromatography, M: PageRuler Prestained Protein Ladder (Fermentas). **(B)** Superimposed chromatograms of Imu3 protein monomers (darker line) (HPLC, size-exlusion) with absorption values at 280 nm normalised. LexA protein self-cleavage products were used as standards (lighter line).

**Figure 2:**
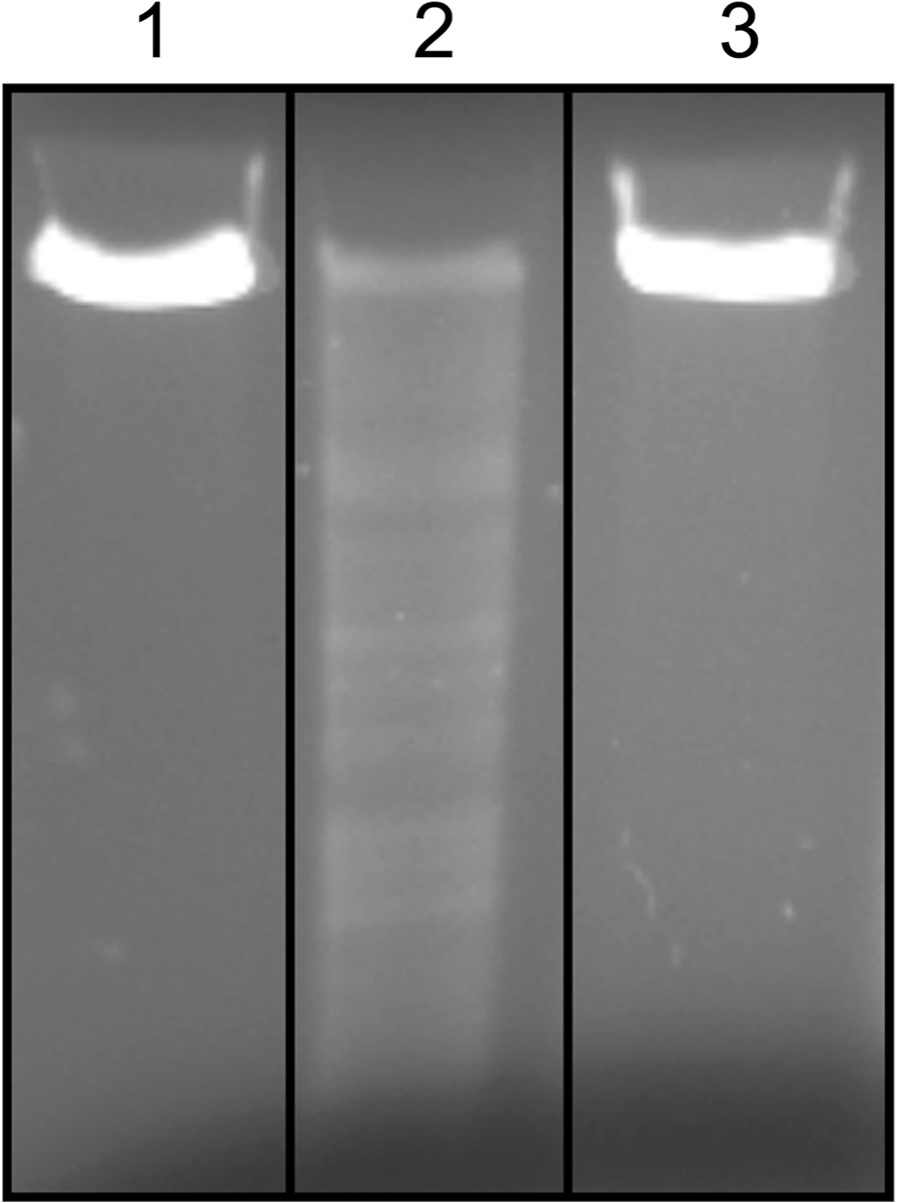
**Imu3 protection against colicin E7 DNase activity.** Lane **1** shows linear dsDNA, lane **2** colicin E7 with linear dsDNA, and lane **3** linear DNA with colicin E7 activity and Imu3.

Isolation of Immunity protein 3 (Imu3) with Ni-NTA affinity chromatography provided protein fractions with appropriate purity; (Figure 1A). DNA binding ability was not affected by the presence or absence of the his-tag, as both precipitated linear DNA (Additional file 1: Figure S1). The theoretical and actual mass (11.497 kDa) of the purified Imu3 differed by 1.5 Da (measured by ESI + and Q-Tof; Waters-Micromass, United Kingdom, data not shown), indicating that Imu3 is not post-translationally modified. Parret and DeMot [5] previously described an approximately 45% sequence identity of the C-terminal region of the Usp protein with known nuclease colicins, such as colicins E7 and E9. Although it has been shown that colicin E7 and its immunity protein form a high-affinity complex [11], we were not able to confirm the formation of a high affinity complex between Usp and any of the three smaller proteins encoded downstream of the *usp* gene (data not shown) which were previously proposed to protect the Usp-producing cell against its endonucleolytic activity [5]. Nevertheless, our results showed that Imu3 protects isolated DNA from digestion by the nuclease colicin E7, indicating a nonspecific protection mechanism that is distinct from that of the colicin immunity proteins (Figure 2).

**Figure 3:**
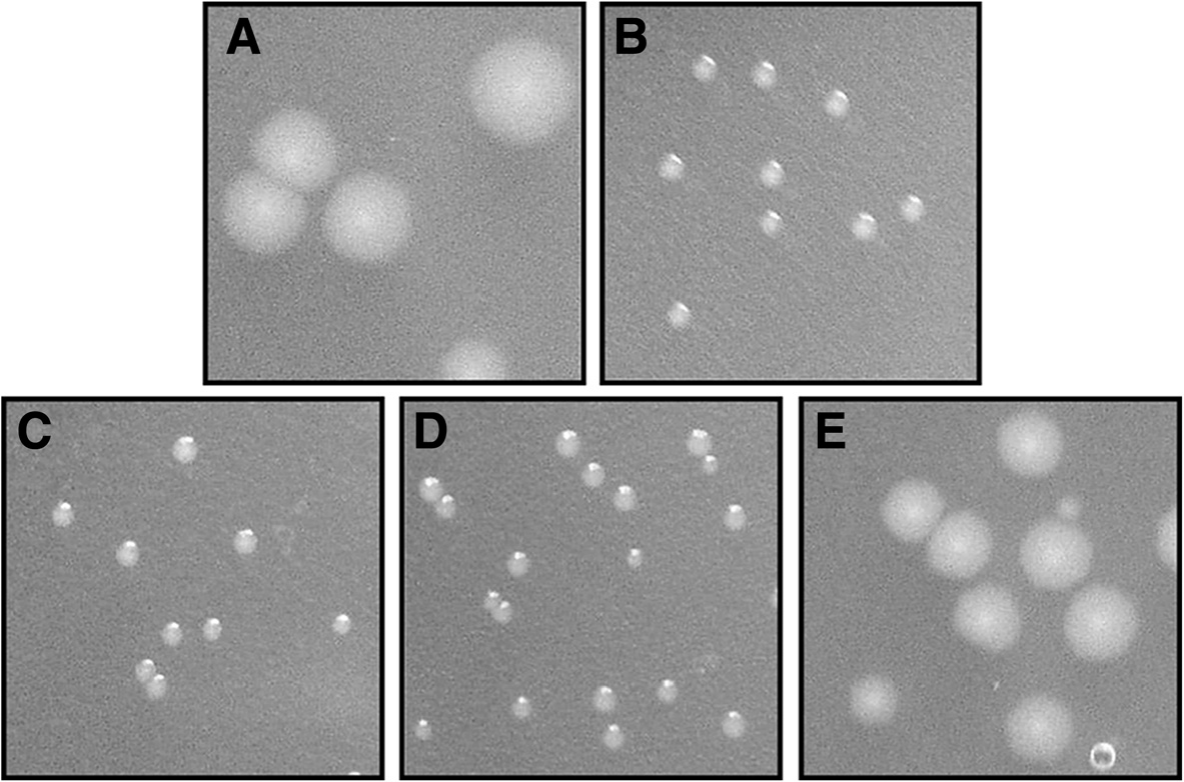
**Protection of *E. coli *Usp producing cells by Imu proteins.** Colonies encoding: **A)***usp imu1*, *imu2* and *imu3*, **B)** only *usp***C)***usp imu1*, **D)***usp imu2,* and **E)***usp imu3* gene. The concentrations of the plated transformation mixtures were adjusted to obtain a comparable number of transformants for each strain.

Induction of the cloned *usp* gene (without the immunity protein genes) was either lethal (liquid media) or resulted in severely diminished growth (plates). Of the three potential immunity proteins, when cloned separately downstream of the *usp* gene, Imu3 showed the greatest degree of protection as the number of transformants obtained was repeatedly higher, with larger colonies than for the other two (Figure 3, Table 1). We therefore focused our further investigation on Imu3.

**Table 1:** **Protection of Usp producing *E. coli *** **by the individual Imu proteins**

**Strain**	**% of transformants relative to control (*usp***^***+ ***^ ***imu1-3*)**
*usp*^*+*^	1.7 ± 1.2
*usp*^*+*^*imu1*	2.4 ± 1.2
*usp*^*+*^*imu2*	4.1 ± 2.0
*usp*^*+*^*imu3*	10.6 ± 4.0

Relative numbers of transformants obtained with plasmids carrying the *usp* gene without and with the individual *imu* genes.

### Imu3 dimerisation and USP binding

Imu3 has fairly high sequence similarity to the colicin E7 immunity protein Cei, approximately 66% sequence identity as established with the MEGA program package, which was previously reported to form monomers [12]. We investigated potential dimer formation by Imu3, using the cross-linking glutaraldehyde assay, native PAGE electrophoresis and size exclusion chromatography (HPLC). Native PAGE as well as HPLC experiments clearly showed that, Imu3 does not form dimers or multimers since a single peak of size between 11 and 13 kDa was observed regardless of the presence or absence of DNA (Figure 1B). Cross-linking studies of equimolar mixtures of Imu3 and Usp also showed no complex formation (Additional file 2: Figure S2).

### DNA/RNA binding

**Figure 4:**
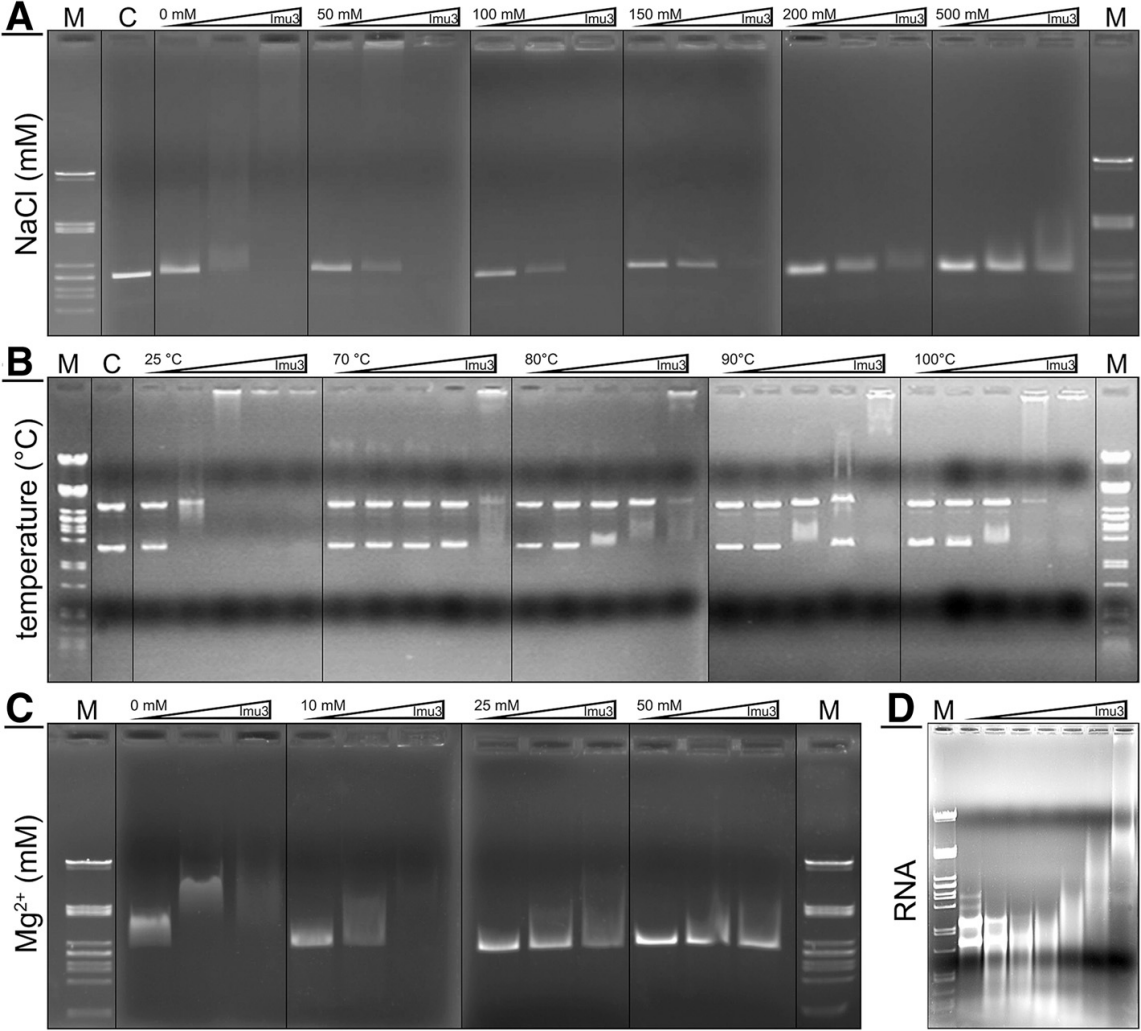
**Representative electromobility shift assays on 0.8% agarose gels.** Effects of Imu3 binding to DNA (“a” and “c” with pUC19/*Eco*RI, “b” with pUC19 and “d” RNA). **(A)** DNA: effect of NaCl (0 to 500 mM), Imu3 concentrations 0.3, 0.6 and 1.2 μg. **(B)** DNA: effect of temperature (10-min incubation), Imu3 additions 0.0625 to 1.0 μg (two fold increase/step). **(C)** DNA: effect of Mg^2+^ ions, Imu3 concentrations 0.3, 0.6 and 1.2 μg. **(D)** RNA: Imu3 additions 0.312 to 10 μg (two fold concentration increase/step). **(A, B, C, D)** M: λ/*Pst*I DNA marker; C: control (pUC19/*Eco*RI alone).

Our data thus indicate that the Usp-producing cell is protected from the DNase activity of its own Usp by a mechanism that is distinct from that of colicin-producing cells. Surprisingly, EMSA showed that Imu3 binds linear and circular (Figure 4B) DNA as well as RNA molecules. When Imu3 reached a critical concentration (*ca*. 1 μg Imu3 per 100 ng double-stranded linear or circular DNA), it repeatedly precipitated the DNA, which resulted in total retardation/precipitation of DNA in the electrophoresis (Figure 4A). When Imu3 was subjected to treatment with increasing concentrations of ions (NaCl or Mg^2+^), the effects of DNA retardation were decreased (Figure 4A and C). Incubations at higher temperatures (70-100°C) also reduced the gel shift effects of Imu3 on DNA (Figure 4B). The EMSA studies with DNA or *E. coli* total RNA clearly showed that Imu3 has DNA-binding as well as RNA-binding abilities. No such activity was observed with Imu1 or Imu2 (data not shown).

**Figure 5:**
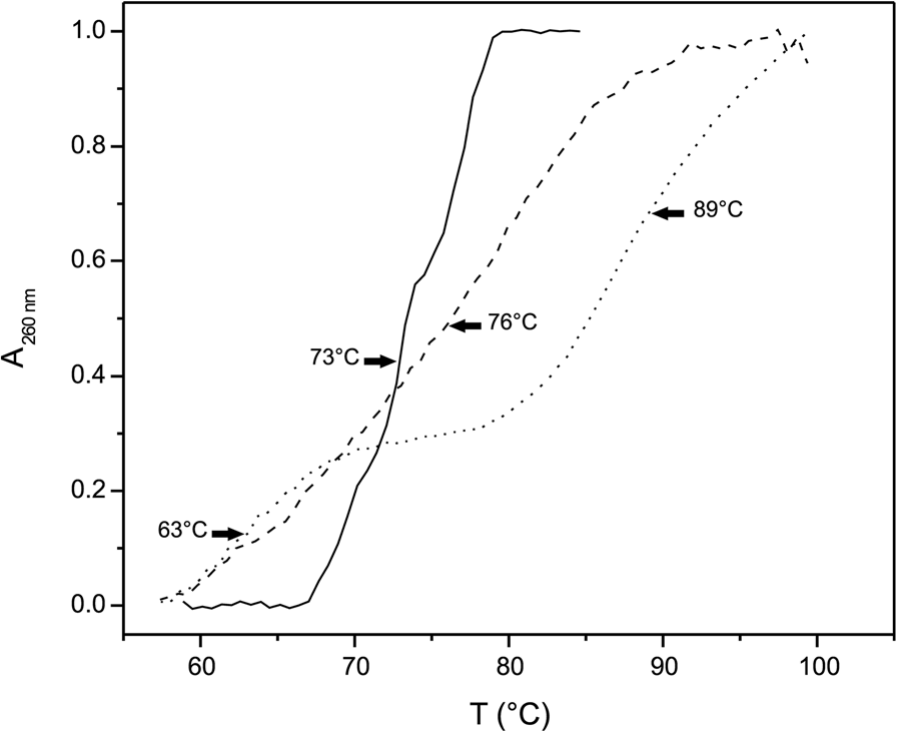
**Thermal denaturation curves of 100 ng pUC19/*Eco *RI DNA.** DNA alone (solid line); DNA with Imu3 at 0.3 μg (dashed line) and 1.0 μg (dotted line). Signal of Imu3 alone was subtracted where necessary, and all curves were normalised. The arrows indicate the Tm values.

Furthermore, thermal denaturation curves (A_260_) showed a stabilising effect of Imu3 on the linear double-stranded DNA molecule. The melting temperature (determined graphically) of DNA alone was 73°C, which increased by 3°C (T_m_ = 76°C) when an aliquot of 0.3 μg Imu3 was added in the EMSA studies. The DNA melting temperature was further raised by an additional 13°C (T_m_ = 89°C) when a 1 μg aliquot of Imu3 was added. This concentration of Imu3 saturated the DNA, and the melting curve revealed a two-phase thermal transition. One transition showed a stabilisation effect (89°C), whereas the other transition (at 63°C) was shown to be destabilising (in terms of thermal stability), most probably due to partial DNA precipitation (Figure 5).

### Minimal DNA length for Imu3 binding

**Figure 6:**
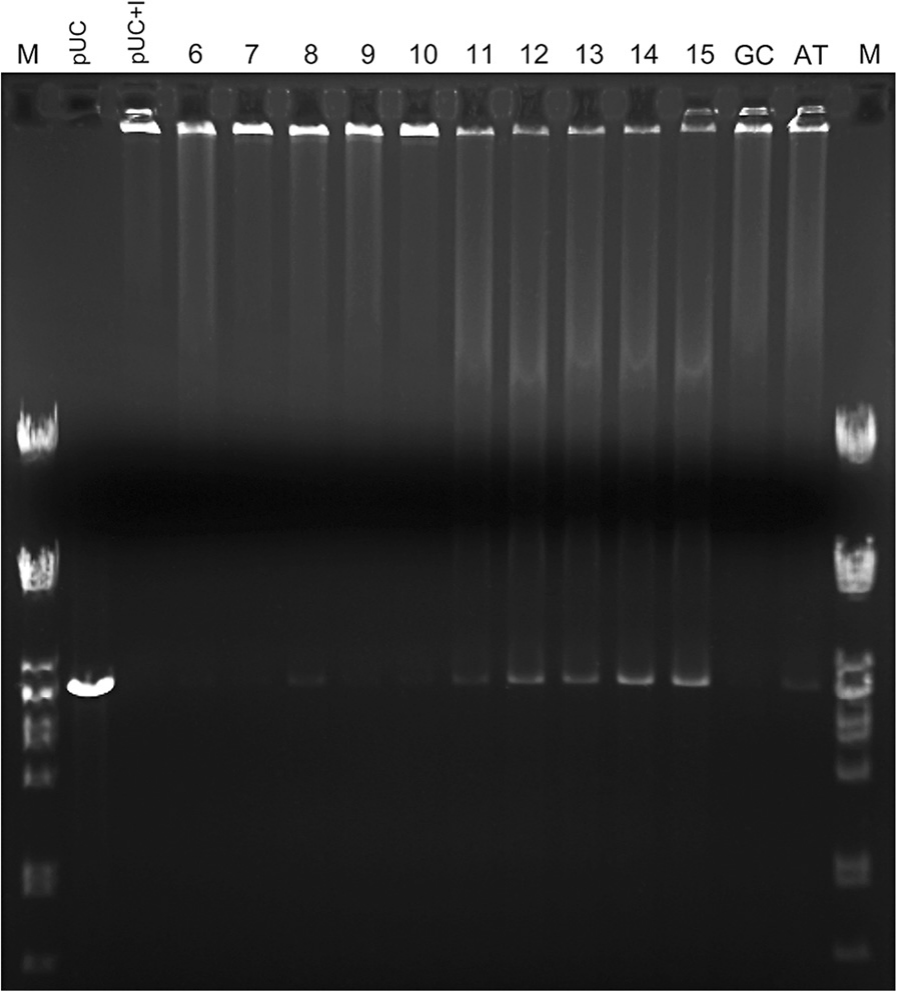
**Electromobility shift assay with short DNA fragments on 0.8% agarose gel.** pUC19, plasmid alone; pUC19 + I, plasmid with Imu3 protein. Lane numbers correspond to number of bases in single-stranded DNA oligonucleotides used (i.e. 6, 7, 8, 9, 10, 11, 12, 13, 14 and 15 bases long). Lanes 6-15, after incubation of Imu3 and corresponding oligonucleotide, 100 ng linear pUC19/*Eco*RI DNA (target) was added. M: λ/*Pst*I marker.

Binding of short DNA fragments to Imu3 occupied all its free DNA binding sites, and therefore prevented subsequent binding of Imu3 to indicator DNA (*Eco*RI linearised pUC19). These EMSA tests showed that free Imu3 starts to bind to oligonucleotides longer than 11 base pairs, observed as the reappearance of unbound indicator DNA (absence of precipitation). These results indicate that 11 base pairs is the minimal DNA length required for Imu3 binding (Figure 6).

EMSA tests with short double stranded DNA fragments (re-annealed oligonucleotides) were also performed however, the results were inconclusive since we repeatedly observed the recurring effect of unbound Imu3 that re-/dis-appeared every 3-5 nucleotides of the oligonucleotide length; however, the underlying basis of this phenomena is unclear.

### Separation of Imu3 from DNA and subsequent DNA integrity analysis

Separation of the DNA-Imu3 complex, was examined under different conditions. Exposure of the DNA–Imu3 complex to pH values between pH 3 and pH 13 showed separation at values between pH 11 and pH 12. However, at pH values higher than pH 12.5, DNA degradation was also observed. When the DNA–Imu3 complex was heated to 100°C for 5 min in the presence of different NaCl concentrations, separation of Imu3 from DNA was observed at 0.5 M NaCl or higher (Additional file 3: Figure S3). Incubation of Imu3-DNA complexes with proteinase K resulted in unbound DNA due to degradation of Imu3.

**Figure 7:**
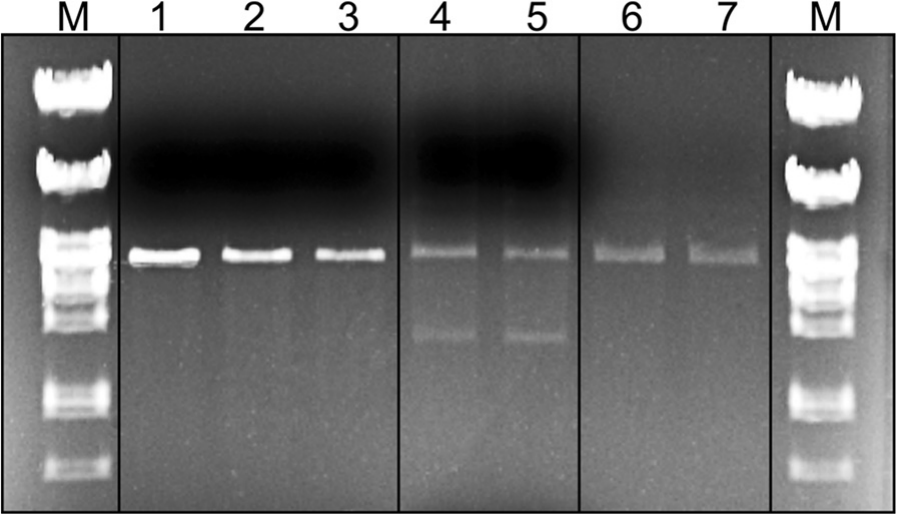
**Representative electromobility shift assays of re-ligated DNA previously complexed with Imu3 (0.8% agarose gels).** Lane 1, 100 ng pUC19/*Eco*RI DNA; lane 2: 100 ng pUC19/*Eco*RI DNA purified with the QIAprep kit; lane 3: 100 ng pUC19/*Eco*RI DNA–Imu3 complex purified with the QIAprep kit; lane 4: ligation reaction of purified DNA; lane 5: ligation reaction of purified DNA–Imu3 complex; lane 6: restriction (*Eco*RI) of ligation reaction of purified DNA (from lane 4); lane 7: restriction (*Eco*RI) of ligation reaction of purified DNA–Imu3 complex (from lane 5). M: λ/*Pst*I marker.

To determine whether DNA exposed to Imu3 could subsequently be used for molecular biological manipulations, linear plasmid pBR322 DNA that had been previously complexed with Imu3 was purified with the QIAgen commercial kit. This DNA could be re-ligated, transformed into *E. coli*, and again subjected to restriction enzyme activity. The integrity of precipitated and religated plasmid DNA was confirmed on the basis of expression of the ampicillin resistance gene among 500 analysed transformants (described in Methods). All procedures were also performed with DNA that had not been previously complexed as a control, and no apparent losses in quantity or quality of DNA were observed (with exception of losses originating from the DNA purification procedure) (Figure 7). Further, we found that Imu3 precipitated DNA from highly (1.5 × 10^-4^ fold) diluted solutions, where 1 μL (100 ng) of linear plasmid DNA was diluted in 15 mL. This procedure yielded less DNA as the control but could without doubt be optimised with appropriate protocol modification (Additional file 4). The colicin DNases and their cognate immunity proteins are known to form high affinity complexes with the DNase domain [11,12]. In the present study, despite its two preserved histidines, as nuclease inactivation motifs that are present throughout the DNase immunity protein family, Imu3 showed no coupling with the USP protein, and Imu3 alone was shown to be sufficient for protection of Usp-producing cells. Not unexpectedly due to the sequence similarity of Imu3 with the colicin E7 immunity protein, which was shown by Dennis et al. [12] to be monomeric, we demonstrated, on the basis of different experiments that Imu3 does not undergo dimerisation or multimerisation.

To the best of our knowledge, no known functions have been described yet for the protein products of *orfU1*, *orfU2* and *orfU3* (here referred to as Imu1, Imu2 and Imu3). Although all three immunity proteins of the Usp cluster share a high degree of sequence similarity, the present study shows that Imu3 has a distinct RNA- and DNA-binding ability. Nevertheless, additional as of yet unresolved mechanisms could be involved in protection of Usp producing cells by its cognate immunity proteins. Interestingly, protein-mediated DNA precipitation has been reported in studies describing eukaryotic histones and the *E. coli* global regulator, protein HU, a known DNA-binding protein [13,14]. Operons, such as those of colicins, that encode proteins that can be detrimental to the producing cell are regulated precisely to ensure appropriate timing of synthesis and avoid untimely death of the producer [15-17]. We can thus speculate that synthesis of Usp and its associated Imu1-3 proteins could also be tightly regulated, limiting their production to avoid overt degradation and masking of the producers’ genome. Indeed high expression levels of *imu3* (IPTG induced for protein isolation) are toxic for producing cells. DNA-binding (basic) proteins usually have an overall positive charge that facilitates their binding to DNA. The Imu3 protein, has a theoretical isoelectric point of *ca*. 4.4, which implies that the DNA-binding region must be localised only on part of the tertiary structure of the molecule. Different online DNA-binding motif search tools were used to identify a potential Imu3 DNA binding motif [18,19]. The results imply that the DNA-binding ability of Imu3 probably originates from the helix-turn-helix motif.

## Conclusions

In conclusion, our study shows that Imu3 like the colicin E7 immunity protein Cei, does not form dimers and in addition, does not form a tight complex with the Usp protein. However, in contrast to the two other small proteins of the Usp pathogenicity island, Imu1 and 2, Imu3 does bind DNA and RNA. We propose that Usp producing cells are protected from genome fragmentation by Imu3 DNA masking. Further, as Imu3 precipitates but does not damage DNA we believe that could have biotechnological potential.

## Methods

### Plasmid construction and protein expression

The nucleotide sequences that encode Imu3 (USP-associated immunity protein 3 from *E. coli*) were amplified from the genomic DNA of the uropathogenic *E. coli* strain TA211 using standard PCR reactions. The Imu3n-F 5′-TTT*CTCGAG*CTATAATTTTAAAGATGAAATAG-3′ and Imu3n.R 5′-TTT*ACGCGT*TATTTAGAGTCTTTAAACAAG-3′ primers were used, with *Xho*I and *Mlu*I restriction sites, respectively (in italics). The PCR product was cloned into the blunted pJET1.2 plasmid (Fermentas), and Usp-coding sequences were subsequently excised and re-cloned into the *Xho*I and *Mlu*I sites of the pET8c expression plasmid, with an N-terminal His tag (Novagen). Subsequently, Imu3 was expressed in the *E. coli* strain BL21(DE3) pLysE as described by the manufacturer (QIAgen). Briefly, an overnight culture of *E. coli* BL21(DE3) pLysE was diluted in liquid Luria Bertani medium supplemented with 120 mg/L ampicillin (LBAp medium) to an OD_600_ of 0.05 at 37°C, and grown to an OD_600_ of 0.6. Imu3 production was induced by the addition of isopropyl β-D-1-thiogalactopyranoside (IPTG) to 0.8 mM final concentration. The culture was grown for an additional 4 h, and then the biomass was collected by 10 min centrifugation at 4,000× *g*.

All of the isolation steps were carried out at 4°C. The collected biomass was treated with DNase, RNase and lysozyme on ice for 1 h, as described by the manufacturer (QIAgen), and complete EDTA-free protease inhibitor cocktail (Roche) was added. The cells were ruptured with 12 consecutive ultrasonication bursts (alternating 30 s pulse, 30 s pause) at the 55 setting (Sonics Vibra Cell). The cell lysates were cleared by three 20 min centrifugations at 20,000× g. All of the other protein isolation steps were carried out. When needed, Imu3 was further purified with size-exclusion FPLC chromatography (Superdex 75 HR 10/30, Amersham Biosciences) equilibrated with 50 mM Tris-HCl, pH 7.5, containing 0.15 M NaCl. Buffer exchanges were carried out using Amicon MWCO 3 kDa microconcentrators (Millipore). The his-tag was removed with the Thrombin Cleavage Capture Kit (Novagen) as described by the manufacturer. Actual mass of Imu3 protein was determined via mass spectrometry ESI + and Q-Tof (Waters-Micromass, United Kingdom).

The degree of Usp-producing cell protection provided by each of the three individual immunity proteins (Imu1-3) was examined in *E. coli* BL21(DE3) pLysE cells that were transformed with the plasmid pET8c carrying the combination of Usp and either Imu1, Imu2 or Imu3. The transformants were isolated on LB Ap plates with IPTG (0.8 mM final concentration) after being grown overnight at 37°C.

### Imu3 and Usp binding

Formation of a Imu3 dimer was checked using the cross-linking glutaraldehyde assay as previously described [20], native PAGE and size exclusion chromatography (HPLC).

Imu3 samples (2 mg/mL) with or without the addition of 2.7 kbp double-stranded linear DNA (pUC19/*Eco*RI) were initially incubated at 37°C for 30 min, to allow for potential multimerization. Samples were then subjected to either native PAGE resolution or to the glutaraldehyde cross-linking procedure and SDS-PAGE resolution, with the LexA protein as a dimerisation-positive control. Aditionally, Imu3 was checked for dimerisation with size exclusion chromatography (HPLC, Phenomenex Biosep SEC-S2000 column, flow rate: 1 mL/min, 50 mM NaH_2_PO_4_, 300 mM NaCl, pH8), self-cleaved LexA protein was used as a standard (11 kDa, 13 kDa and 26 kDa).

Formation of the Imu3–USP complex was also investigated using the glutaraldehyde assay, after Imu3 and Usp had been mixed in equimolar ratios.

### DNA/RNA binding

Various concentrations of either *Eco*RI linearised pUC19 DNA or total RNA (isolated from *E. coli*) and the Imu3 protein were used to establish the nucleic-acid-binding ability of Imu3. The Imu3 was incubated with either the DNA or RNA in TE buffer (10 mM Tris, 1 mM EDTA, pH 8) at 37°C for 30 min, prior to the electromobility shift assays (EMSAs) with 0.8% agarose gels. The effects of temperature (10 min incubation at 70°C, 80°C, 90°C and 100°C), pH (6, 7, 8, 9), NaCl (0-500 mM) and Mg^2+^ ions concentrations (0-50 mM) on the DNA binding ability of Imu3 were studied.

Thermal denaturation curves of linearised pUC19 DNA and the Imu3 protein were carried out in 5 mM cacodylic buffer (pH 6.5) using a UV-vis spectrophotometer (Cary Varian Cary 100 Bio, Australia) equipped with a thermoelectrically controlled cell holder. UV absorption was measured as a function of temperature (UV melting curves) for different ratios of linear DNA and Imu3 (0, 0.3 and 1.0 μg per 100 ng DNA), at 260 nm. The UV melting temperature ranged from 25°C to 99°C, with a heating rate of 1°C•min^-1^ and an equilibration time of 1 min. The melting curves of buffer and of the Imu3 protein alone were subtracted from the melting curves of the DNA–Imu3 protein complex, providing curves that show only the changes in the thermal stability of the DNA.

Further, the influences of pH, temperature and ionic strength on the separation of the DNA–Imu3 complex were examined. The effects of pH, were examined in the range from pH 3 to pH 13. Buffers used for these pH values were the following: pH 3-5, citric buffer; pH 6, MES buffer; pH 7-9, TRIS buffer; pH 10-12, glycine/NaOH buffer; pH 13, NaOH. The impacts of various ions on the separation of the DNA–Imu3 complex were studied as 0-1 M NaCl, 350 mM KCl, 350 mM NaSCN, 70 mM MgCl_2_, 0.7% SDS, 1-3 M (NH_4_)_2_SO_4_ and 2.3 M guanidinium thiocyanate. The effects of temperature were studied 80°C and 95°C, with a 10 min incubation of the complex, and at 100°C, with a 5 min incubation.

To examine whether Imu3 binding to DNA triggers any DNA damage, religation experiments were performed. Initially, the linear plasmid DNA (pUC19) was incubated with the Imu3 protein at 37°C for 30 min, to allow for the DNA–Imu3 complex to form. The samples were subsequently purified using the QIAprep Spin Miniprep kits (QIAgen). To check DNA integrity, the linearised DNA was used for a (self) ligation reaction (Fermentas); half of the ligation mixture was transformed into *E. coli* DH5α, while the other half was subjected to a second restriction (*Eco*RI).

The structural integrity of the Imu3 precipitated plasmid DNA was also investigated on the basis of detection of potential mutations within a non-selected marker, the ampicillin resistance gene. For this purpose, plasmid pBR322 carrying both tetracycline and ampicillin resistance genes was employed. Plasmid DNA was digested with *Pst*I, with a single restriction site within the ampicillin resistance gene to yield one linear DNA fragment. Following gel electrophoresis the linear plasmid DNA was precipitated with Imu3 and centrifuged for 10 minutes at 4°C, followed by washing with 0.5 ml of TE buffer. The pellet was subsequently treated with the PCR Cleaning Kit (Thermo Scientific) and several μl of the isolate were employed for re-ligation. In control experiments, ligase was omitted. Transformants were isolated upon selection for tetracycline resistance. Five hundred transformants were further screened and all found to express ampicillin resistance.

### Minimum DNA length for Imu3 binding

The minimum length of single-stranded DNA required for Imu3 binding was studied by initially saturating Imu3 with short DNA fragments of known lengths. The reaction mixtures of Imu3 (10 μg) and an at least 5-fold excess of oligonucleotides, were incubated for 30 min at 37°C (allowing Imu3 to bind to oligonucleotides of sufficient length) prior to the addition of the indicator DNA (100 ng pUC19/*Eco*RI). Oligonucleotides too short to form a complex with Imu3, bind to the indicator DNA provoking an electro-mobility shift. Samples were incubated for another 30 min at 37°C, to allow potential binding of Imu3 to the indicator DNA. The samples were later resolved on 0.8% agarose Tris-borate gels. The short DNA fragments were all synthesised as single-stranded oligonucleotides, the sequences of which are given in Table 2.

**Table 2:** Oligonucleotide sequences used to determine the minimal length of single-stranded and double-stranded DNA for DNA–Imu3 complex formation

**Oligonucleotide**	**5′-3′ sequence**	**gCc**
6-mer (M13)	gCggTTcv	67
7-mer (M13)	gCggTTC	63
8-mer (M13)	TggCggTT	63
9-mer (M13)	gTggCggTT	67
10-mer (M13)	ggTggCggTT	70
11-mer (M13)	ggTggCggTTC	73
12-mer (M13)	AgggTggCggTT	67
13-mer (M13)	gAgggTggCggTT	69
14-mer (M13)	gAgggTggCggTTC	71
15-mer (M13)	gAgggTggCggTTCT	67
15-TATA (poly AT)	TATATATATATATAT	0
15-gCgC (poly GC)	gCgCgCgCgCgCgCg	100

The lengths of the oligonucleotides spanned from 6 to 32 nucleotides.

## Competing interests

The authors declare that they have no competing interests.

## Authors’ contributions

Conceived and designed the experiments: MČ ZP DŽB. Performed the experiments: MČ MB ZP. Contributed reagents/materials/analysis tools: DŽB. Wrote the paper: MČ DŽB. All authors read and approved the final manuscript.

## Supplementary Material

Additional file 1: Figure S1 Effect of His-tag presence on Imu3 DNA-binding ability. M: PageRuler Prestained Protein Ladder (Fermentas); 1. Imu3 with His-tag (SDS-PAGE); 2. Imu3 with His-tag removed (SDS-PAGE); 3. 100 ng of pUC19/*Eco*RI (agarose electrophoresis, AE); 4. 100 ng of pUC19/*Eco*RI complexed with Imu3 with His-tag removed (AE). Click here for file

Additional file 2: Figure S2 Dimerisation of Imu3 and USP proteins, 10% SDS PAGE gel. M: PageRuler Prestained Protein Ladder (Fermentas); 1. Imu3 protein (11.5 kDa); 2. USP protein (67 kDa). Samples following cross-linking with glutaraldehyde (3-7); 3. Imu3 protein, 4. USP protein, 5. Imu3 and USP, 6. Imu3 and USP with addition of DNA, 7. LexA protein mono/dimer (24/48 kDa). Click here for file

Additional file 3: Figure S3 Representative electromobility shift assays on 0.8% agarose gels. Effects of pH, NaCl and temperature on DNA–Imu3 complex relaxation. Lane 1: pUC19/*Eco*RI DNA (100 ng); lane 2: Imu3-pUC19/*Eco*RI untreated complex; lanes 3-7: Imu3-pUC19/*Eco*RI complex treated with pH values 10, 11, 12, 12.5, 13; lanes 8-11: Imu3-pUC19/*Eco*RI complex incubated 5 min at 100°C in the presence 0, 0.2, 0.5 and 1 M NaCl; lanes 12-14: complex treated with 0, 0.5 and 1 M NaCl at pH 12. Click here for file

Additional file 4: Figure S4 DNA precipitation from diluted systems. On agarose gel: 1. pUC19/*Eco*RI (100 ng); 2. pUC19/*Eco*RI purified with GeneJet PCR purification Kit (Fermentas); 3. 100 ng of pUC19/*Eco*RI diluted 1.5 × 10^-4^ in 15 mL buffer and salvaged with Imu3 precipitation and subsequent GeneJet PCR purification Kit Imu3 removal. Click here for file
