# Supplementary figures and images for: The Escherichia coli uropathogenic-specific-protein-associated immunity protein 3 (Imu3) has nucleic acid -binding activity
Source: BMC Microbiol. 2014 Jan 28;14:16. doi: 10.1186/1471-2180-14-16 (PMC3917654; doi:10.1186/1471-2180-14-16)

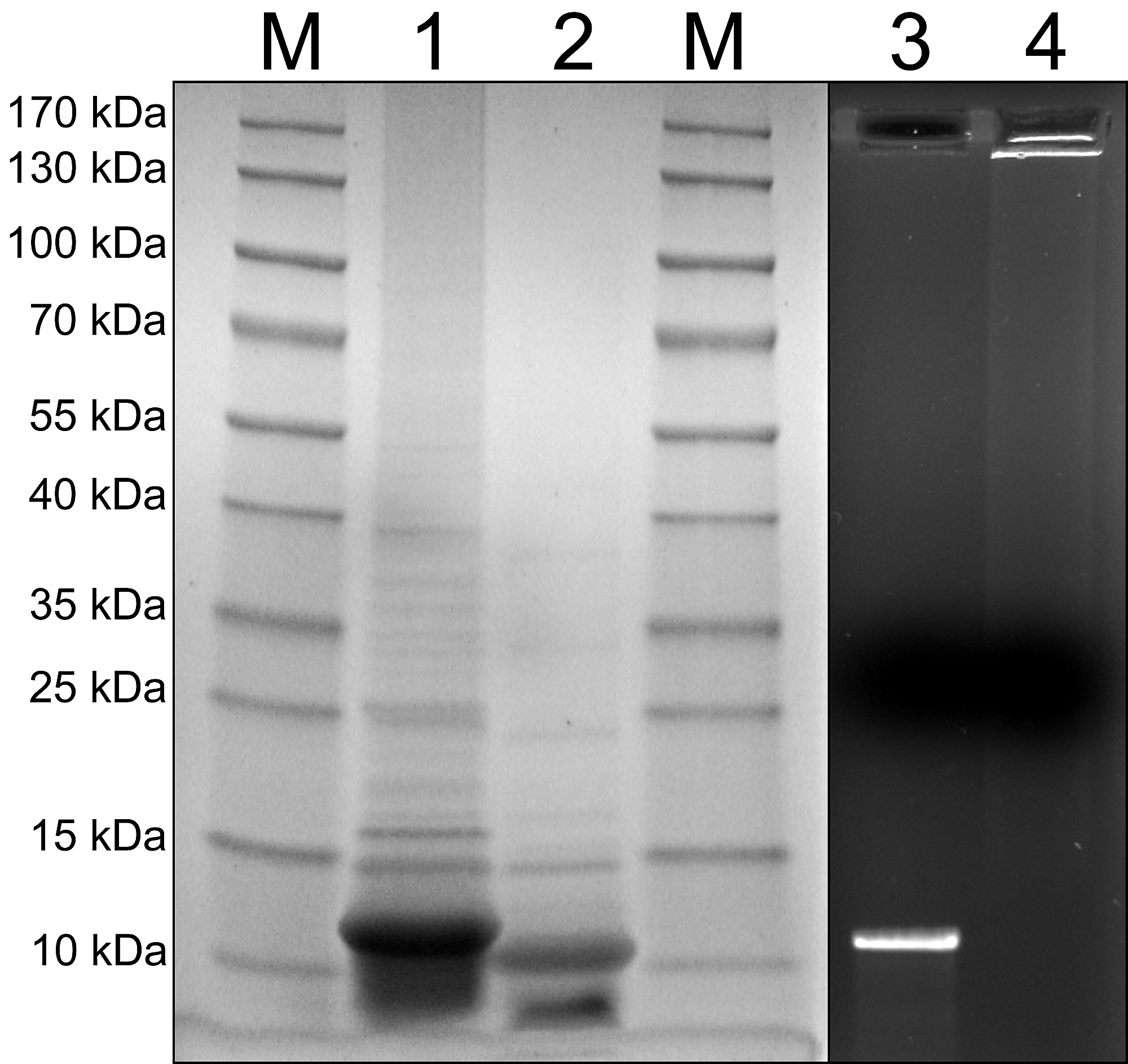

Supplement: Additional file 1: Figure S1 — Effect of His-tag presence on Imu3 DNA-binding ability. M: PageRuler Prestained Protein Ladder (Fermentas); 1. Imu3 with His-tag (SDS-PAGE); 2. Imu3 with His-tag removed (SDS-PAGE); 3. 100 ng of pUC19/EcoRI (agarose electrophoresis, AE); 4. 100 ng of pUC19/EcoRI complexed with Imu3 with His-tag removed (AE). [file 1471-2180-14-16-S1.jpeg]

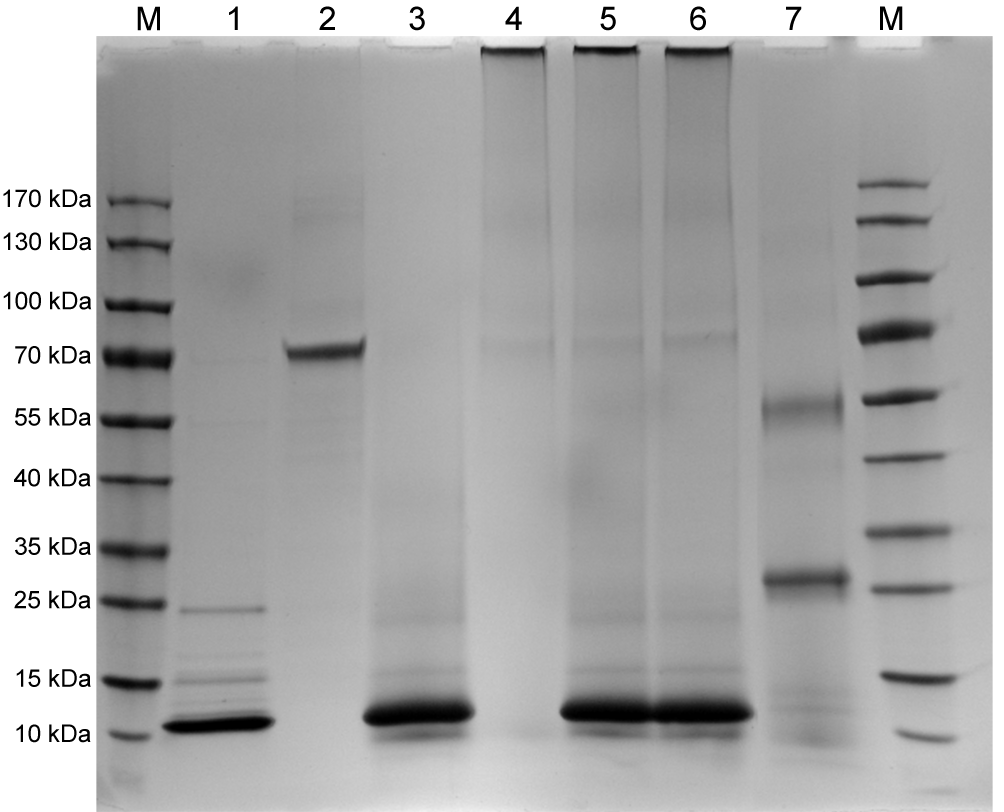

Supplement: Additional file 2: Figure S2 — Dimerisation of Imu3 and USP proteins, 10% SDS PAGE gel. M: PageRuler Prestained Protein Ladder (Fermentas); 1. Imu3 protein (11.5 kDa); 2. USP protein (67 kDa). Samples following cross-linking with glutaraldehyde (3-7); 3. Imu3 protein, 4. USP protein, 5. Imu3 and USP, 6. Imu3 and USP with addition of DNA, 7. LexA protein mono/dimer (24/48 kDa). [file 1471-2180-14-16-S2.tiff]

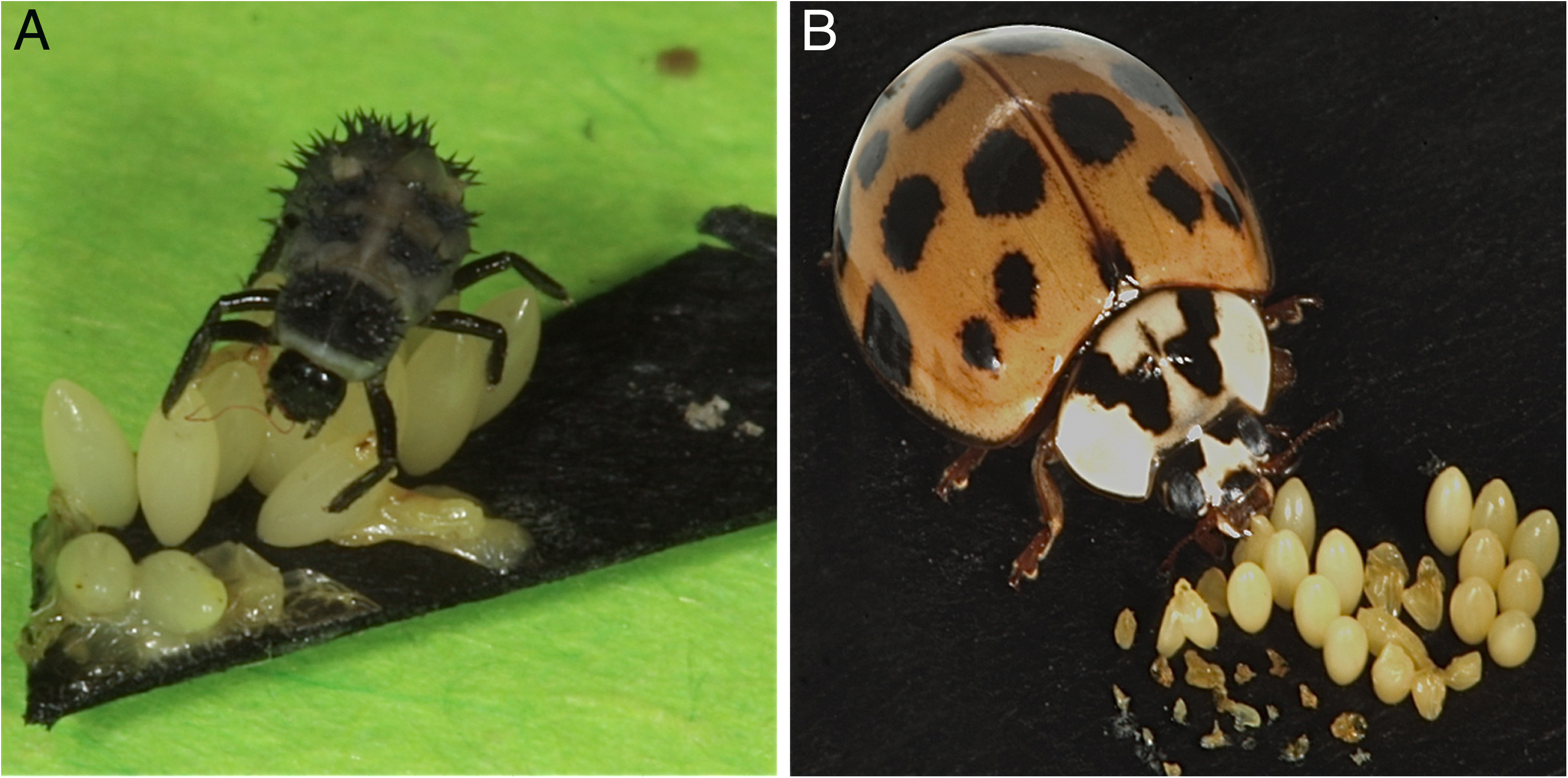

Supplement: Additional file 3: Figure S3 — Representative electromobility shift assays on 0.8% agarose gels. Effects of pH, NaCl and temperature on DNA–Imu3 complex relaxation. Lane 1: pUC19/EcoRI DNA (100 ng); lane 2: Imu3-pUC19/EcoRI untreated complex; lanes 3-7: Imu3-pUC19/EcoRI complex treated with pH values 10, 11, 12, 12.5, 13; lanes 8-11: Imu3-pUC19/EcoRI complex incubated 5 min at 100°C in the presence 0, 0.2, 0.5 and 1 M NaCl; lanes 12-14: complex treated with 0, 0.5 and 1 M NaCl at pH 12. [file 1471-2180-14-16-S3.tiff]

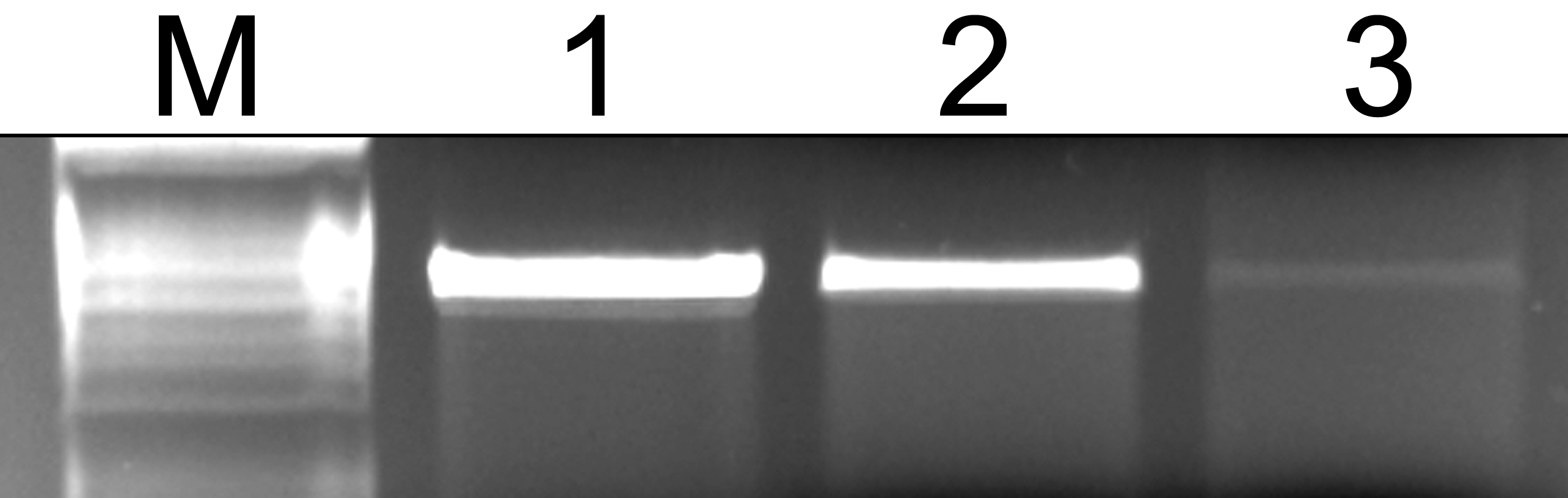

Supplement: Additional file 4: Figure S4 — DNA precipitation from diluted systems. On agarose gel: 1. pUC19/EcoRI (100 ng); 2. pUC19/EcoRI purified with GeneJet PCR purification Kit (Fermentas); 3. 100 ng of pUC19/EcoRI diluted 1.5 × 10^-4^ in 15 mL buffer and salvaged with Imu3 precipitation and subsequent GeneJet PCR purification Kit Imu3 removal. [file 1471-2180-14-16-S4.tiff]
